# PRIME Immunology: Self-directed Introduction to Medical School Immunology

**DOI:** 10.1007/s40670-021-01326-7

**Published:** 2021-06-16

**Authors:** Alessandra G. Tomasi, Thomas Belhorn, Frank C. Church

**Affiliations:** 1 University of North Carolina School of Medicine, Chapel Hill, NC 27599 USA; 2 Mayo Clinic General Internal Medicine, 200 First St. SW, Rochester, MN 55905 USA; 3 Department of Pediatrics, University of North Carolina School of Medicine, Chapel Hill, NC 27599 USA; 4 Department of Pathology and Laboratory Medicine, University of North Carolina School of Medicine, NC Chapel Hill, 27599 USA

**Keywords:** Immunology, Self-paced learning, Online learning, Innate and adaptive immunity, Complement

## Abstract

**Introduction:**

Medical students find immunology difficult to understand and relate to clinically and are often frustrated by the amount of detailed material. We created PRIME Immunology: Preview or Review of Important Material for Everyone: (i) video modules, (ii) Instagram site, and (iii) vocabulary files called Immunology Language.

**Methods:**

The self-paced modules introduced key topics in immunology for students to complete prior to their instructional block.

**Results and Conclusions:**

Use of PRIME Immunology during a 3-year period suggested that providing students with an overview of key topics before the start of their course may (i) reduce student angst about immunology and (ii) improve retention of immunology.

## Background

Coursework in immunology is typically not required for matriculation into most medical schools. Regardless of students’ background, studies show that medical students find immunology difficult to understand and relate to clinically [1, 2]. A source of frustration is an inability to visualize how the immune system works as a whole within the context of pathogen invasion [3]. Due to this often limited and highly variable exposure to the subject prior to medical school, professors and students face a challenge in presenting and learning the material.

Teaching methods that are centered on self-paced or self-directed study may help overcome this challenge [4–8]. As medical education continues to evolve, in many medical schools, basic medical science subjects have become integrated into organ system blocks [9–13]. Medical education institutions using structured integrated curricula have been exploring such methods [14, 15]. Specifically, several studies have demonstrated the efficacy of online modules for medical students, linking their use to improved retention of material and increased effectiveness of subsequent classroom learning [4, 16].

**Fig. 1:**
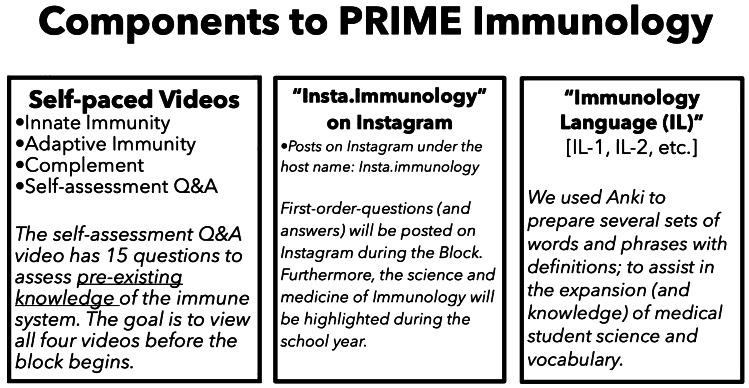
Components of PRIME Immunology. A brief description of the PRIME Immunology curriculum that consists of self-paced videos, Instagram site, and vocabulary files

This project was called PRIME Immunology: Preview or Review of Important Material for Everyone, and it consisted of three components: (i) video modules, (ii) an Instagram site called “Insta.Immunology,” and (iii) Anki-based word files called Immunology Language (described in Fig. 1). For the first component (further reviewed here), we created online self-paced video modules that offered a broad introduction to immunological systems for matriculating medical students. By ensuring that every student, regardless of their prior exposure to the subject, would be familiar with key terminologies underlying the foundations of immunology, we hypothesized that the use of the PRIME Immunology curriculum would improve overall retention and effectiveness of future lectures and assignments.

## Activity

Using Keynote software, we created a standardized set of video modules covering key topics in immunology to provide an effective introduction for those with little or no background knowledge in immunology as well as a review for those who had previous education in the field. We created individual modules on innate immunity, adaptive immunity, and the complement system. These three videos culminated in a final multiple-choice self-assessment module. Students were instructed to complete the PRIME Immunology video modules prior to the first day of their immunology instructional block.

The innate immunity module covered physical barriers to infection (anatomic, chemical, and microflora), major cell types involved in innate immunity (granulocytes, monocytes and macrophages, dendritic cells, and natural killer cells), the role of toll-like receptors (TLRs) in recognizing pathogen associated molecular patterns (PAMPs), and cytokine signaling. In the adaptive immunity module, key features of cell-mediated and humoral immunity were discussed, including the major functions and classes of T cells and B cells, plus comparisons in terms of production, differentiation, and specific roles in intracellular and extracellular infection. Lastly, a complement system video introduced the alternative, lectin, and classical pathways, as well as the overall role of complement in response to infection. For the self-assessment at the end, students were instructed that a score of at least 12 out of 15 indicated sufficient preparedness for their immunology course. An answer key was provided with explanations. The self-paced PRIME videos can be viewed here: https://bit.ly/3qUyYv8.

## Results and Discussion

First-year students were surveyed over the course of 3 years to assess their level of exposure to immunology prior to medical school. Of the students who responded, 17% (*n* = 22) had taken at least one course in immunology, defined as > 5 lectures on the subject, while 46% (*n* = 57) had received 5 or fewer lectures on the material (data not shown). A total of 38% (*n* = 47) had no exposure to the field at all (data not included).

Students were surveyed on their use and efficacy of PRIME Immunology at the end of their immunology block in years 2017–2019. Of those who responded, 82% (*n* = 101) watched the videos and completed the self-assessment prior to the start of their course (data not shown).

**Fig. 2:**
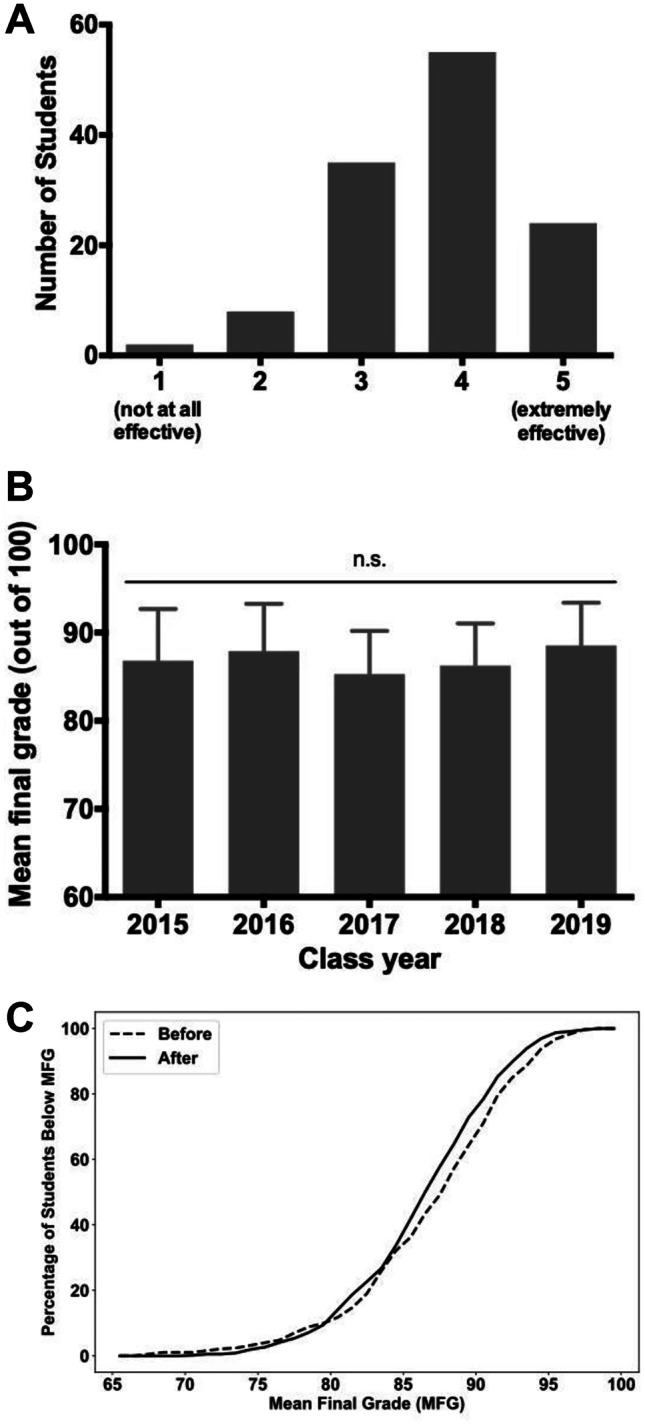
Assessment of the PRIME Immunology curriculum. **A** Student reported efficacy of PRIME Immunology curriculum. When asked to rate their satisfaction with the modules on a 5-point Likert scale (1 = “not at all effective,” 5 = “extremely effective”), 20% of students (*n* = 24) provided a score of 5, 45% (*n* = 55) a score of 4, 28% (*n* = 35) a score of 3, and 6% (*n* = 8) a score of 2. 1% of students (*n* = 2) did not find the modules to be effective at all. **B **Immunology block mean final grade (MFG) by year. Mean final grades for medical students were compared from 2015 to 2019. There was no statistically significant difference among the 5 years, and therefore, no difference in overall class performance before (2015–2016) and after (2017–2019) the introduction of PRIME Immunology. **C **Cumulative distribution of immunology block mean final grade (MFG) by year. Before PRIME (2015–2016, “Before”), four students did not receive a passing score (defined as ≥ 70%) for the immunology instructional block, whereas using PRIME Immunology (2017–2019, “After”), no students received a failing grade

We asked students to rate the efficacy of PRIME Immunology in navigating their immunology block (Fig. 2A). These results suggest the majority of students found these supplemental learning modules positively impacted their learning. A limitation of our study was a low rate of survey completion, with fewer than half of medical students responding. Nevertheless, our findings support other studies in undergraduate medical education in which both students and faculty have found multimedia e-learning to enhance teaching and learning due to accessibility, increased content standardization, and ease of use [17, 18].

Final course numerical grades for students the 2 years before and 3 years during the use of PRIME Immunology were compared to help assess whether introduction of this curriculum had any effect on overall class performance or failure rates. Data from the 2 years immediately preceding the use of PRIME Immunology (2015 and 2016) showed a mean final block grade of 86.8 and 87.96, respectively (Fig. 2B). Data from the first three semesters of using PRIME Immunology (2017, 2018, and 2019) showed a mean final block grade of 85.28, 86.24, and 88.5, respectively (Fig. 2B). There was no statistically significant improvement in overall class performance when comparing the pre-intervention and intervention groups (Fig. 2B). The pre-determined numerical cut-off for a passing grade for all instructional blocks is 70 out of 100. In the pre-intervention groups (class years 2015 and 2016), it was noted that four students did not earn a passing grade in the block, whereas in the intervention groups (class years 2017–2019), no students received a failing grade (Fig. 2C). Multiple factors can contribute to student underperformance. However, particularly for a challenging subject such as immunology, the availability of online materials for self-instruction can help make the complexity and breadth of educational content more manageable [18–22].

In summary, we designed an educational curriculum called PRIME Immunology to provide an effective introduction for students with little background in immunology as well as a review for those who had previous education in this field. The curriculum was thought to be effective by many students and may have been especially beneficial for students who may have otherwise been at risk of failing the immunology course. Ultimately, new developments of online tools to improve the effectiveness of teaching and learning for both students and faculty will undoubtedly continue to redefine standards for medical education.
